# Künstliche Intelligenz in der Gesundheitsvorsorge von Kindern und Jugendlichen – Anwendungsmöglichkeiten und Akzeptanz

**DOI:** 10.1007/s00103-025-04096-4

**Published:** 2025-06-30

**Authors:** Janna-Lina Kerth, Anne Christine Bischops, Maurus Hagemeister, Lisa Reinhart, Kerstin Konrad, Bert Heinrichs, Thomas Meissner

**Affiliations:** 1 Klinik für Allgemeine Pädiatrie, Neonatologie und Kinderkardiologie, Medizinische Fakultät und Universitätsklinikum Düsseldorf, Heinrich-Heine-Universität Düsseldorf, Moorenstr. 5, 40225 Düsseldorf, Deutschland; 2 Klinik für Psychiatrie, Psychosomatik und Psychotherapie des Kindes- und Jugendalters, Uniklinik Aachen, Aachen, Deutschland; 3 JARA-Institut Molecular neuroscience and neuroimaging (INM-11), Forschungszentrum Jülich, Jülich, Deutschland; 4 Institut für Neurowissenschaften und Medizin: Gehirn und Verhalten (INM-7), Forschungszentrum Jülich, Jülich, Deutschland; 5 Institut für Wissenschaft und Ethik (IWE), Universität Bonn, Bonn, Deutschland

**Keywords:** Künstliche Intelligenz, Kinder- und Jugendmedizin, Entwicklungsstörungen, Krankheitsprävention, Akzeptanz von Technologie, Artificial intelligence, Pediatric and adolescent medicine, Developmental disorders, Disease prevention, Technology acceptance

## Abstract

Der Einsatz von künstlicher Intelligenz (KI) in der Kinder- und Jugendmedizin bietet vielfältige Möglichkeiten, insbesondere in der Prävention von chronischen Erkrankungen. KI-gestützte Anwendungen wie maschinelles Lernen zur Analyse von Sprach- oder Bewegungsmustern können beispielsweise helfen, frühzeitig die Diagnose von Autismus-Spektrum-Störungen oder motorischen Entwicklungsverzögerungen zu stellen. Zudem unterstützen KI-basierte Systeme die Therapie von Kindern mit Diabetes mellitus Typ 1 durch automatisierte Insulin-Dosierungssysteme (AID).

KI ermöglicht präzisere Diagnosen, personalisierte Therapieansätze und eine Entlastung des medizinischen Personals. Gleichzeitig gibt es Herausforderungen, die den Einsatz von KI betreffen und bedingen, dass nur wenige Anwendungen bislang Einzug in die klinische Routine gefunden haben. Dazu zählen der Schutz sensibler Daten und die Wahrung der informationellen Selbstbestimmung, die Sicherstellung von Diskriminierungsfreiheit, die Transparenz algorithmischer Entscheidungsprozesse sowie die Akzeptanz durch alle beteiligten Gruppen wie Kinder und Jugendliche, Eltern und medizinisches Personal. Alle beteiligten Gruppen sehen potenzielle Fehlentscheidungen, den Verlust persönlicher Interaktionen sowie die mögliche kommerzielle Nutzung von Daten kritisch. Eltern und Fachkräfte betonen die Bedeutung von klarer Kommunikation, Mitbestimmung und Schulungen für ein besseres Verständnis. Zudem mangelt es oft an strukturierten, hochwertigen großen Datensätzen kompatibler Formate, um die KI zu trainieren.

Eine nachhaltige Integration von KI in der Kinder- und Jugendmedizin erfordert groß angelegte klinische Studien, Zugang zu hochwertigen Datensätzen sowie eine differenzierte Analyse ethischer und sozialer Implikationen.

**Zusatzmaterial online:**

Zusätzliche Informationen sind in der Online-Version dieses Artikels (10.1007/s00103-025-04096-4) enthalten.

## Abstract (en)

The use of artificial intelligence (AI) in pediatric and adolescent medicine offers numerous possibilities, particularly in the prevention of chronic diseases. AI-powered applications such as machine learning for the analysis of speech or movement patterns can, for example, help in the early diagnosis of autism spectrum disorders or motor development delays. In addition, AI-based systems support the treatment of children with type 1 diabetes through automated insulin dosing (AID) systems.

AI enables more accurate diagnoses and personalized therapeutic approaches and helps relieve the burden on medical personnel. At the same time, there are challenges associated with the use of AI, which is why only a few applications have so far become part of routine clinical practice. These challenges include the protection of sensitive data and the respect for informational self-determination, ensuring freedom from discrimination, algorithmic transparency, and the acceptance of AI by all involved groups such as children, adolescents, parents, and medical professionals. All stakeholders express concerns about potential misjudgments, the loss of personal interactions, and the possible commercial use of data. Parents and professionals emphasize the importance of clear communication, shared decision-making, and training to promote better understanding. Moreover, there is often a lack of structured, high-quality, large datasets in compatible formats to effectively train AI systems.

A sustainable integration of AI in pediatric and adolescent medicine requires large-scale clinical studies, access to high-quality datasets, and a nuanced analysis of the ethical and social implications.

## Hintergrund

Der Einsatz von künstlicher Intelligenz (KI) hat in den letzten Jahren in zahlreichen Lebensbereichen deutlich zugenommen – insbesondere in der Medizin. Vor allem in der Bilderkennung und bei der Analyse großer Datenmengen, etwa aus elektronischen Patient:innendatenbanken, existieren bereits vielfältige Anwendungsmöglichkeiten, die allerdings bisher nur zögerlich Einzug in die medizinische Versorgung gehalten haben. Der Großteil der Forschung und Entwicklung erfolgt mit Daten erwachsener Patient:innen und ist auf Kinder und Jugendliche nur bedingt oder gar nicht übertragbar. Zudem gilt es zu beachten, dass Säuglinge und junge Kinder zunächst vollständig auf die Fürsorge anderer angewiesen sind, die in diesem Zuge auch über die Nutzung ihrer Daten entscheiden. Mit zunehmendem Alter erlangen Kinder mehr Autonomie und möchten eigenständig Entscheidungen treffen. Für eine erfolgreiche Implementierung KI-gestützter Gesundheitsanwendungen müssen diese nicht nur durch die Kinder und Jugendlichen selbst, sondern auch von Eltern und medizinischem Personal akzeptiert werden [1].

Der Prävention kommt im Kindes- und Jugendalter eine besondere Bedeutung zu, um ein möglichst gesundes Aufwachsen zu ermöglichen. Bei vielen Erkrankungen, insbesondere solchen, die mit Entwicklungsproblemen einhergehen, kann eine frühzeitige Diagnose maßgeblich zu einem verbesserten Outcome beitragen. In Deutschland sind zwar die sogenannten U‑Untersuchungen, also die regelmäßigen kinderärztlichen Vorsorgen, gesetzlich vorgeschrieben, diese liefern jedoch nur punktuelle Untersuchungsergebnisse und weisen zudem mit zunehmendem Alter immer längere Abstände auf. Vor diesem Hintergrund können digitale Gesundheitsanwendungen, die mithilfe von KI große Datenmengen aus verschiedenen Quellen (u. a. Fotos, Videos, Sprachaufnahmen, medizinische Dokumentation) analysieren, eine wichtige Hilfe bei der Krankheitsfrüherkennung sein.

Die Nachfrage nach Gesundheits-Apps, die Eltern Ratschläge zur Entwicklung von Babys und Kleinkindern geben, ist groß. Allein die App „Oje, ich wachse“ hat im App Store von Apple fast 35.000 Bewertungen und ist in 16 Sprachen erhältlich [2]. Die App „Brainprotect“ verspricht, die motorische und kognitive Entwicklung des Kindes bis zum vierten Lebensjahr, inklusive des voraussichtlichen IQs, anhand der Schwangerschaftsdaten vorauszusagen [3]. Die Datengrundlage für die App bildet dabei jedoch ein Datensatz, der an einem Zentrum in den Jahren 1984–1988 gesammelt wurde, sodass die Aussagekraft der Prognosen bei mehr als 30 Jahre später geborenen Kindern zumindest fraglich ist [4]. Derart wichtige Informationen zu der Datengrundlage sollten auf der Homepage zur App und in der App-Beschreibung transparent für die Nutzer:innen dargelegt werden. Außerdem sollte auf Limitationen bei den Vorhersagen hingewiesen werden. Gleichzeitig zeigt dieses Beispiel die Wichtigkeit der Generierung und Bereitstellung belastbarer Daten.

In der Betreuung von Kindern und Jugendlichen, bei denen bereits eine chronische Erkrankung diagnostiziert wurde, können KI-Anwendungen ebenfalls eine sinnvolle und wichtige Hilfe im Alltag sein. So sind aktuell für viele dieser Patient:innen regelmäßige, engmaschige ärztliche Vorstellungen notwendig, die, je nach Wohnort und Erkrankung, auch eine weite Anreise zu einem spezialisierten Zentrum erfordern. Die Belastung durch eine chronische Erkrankung kann sich auch negativ auf die psychische Gesundheit auswirken [5, 6]. „Symptomtracker“ können mit regelmäßigen Befragungen oder Datenerhebungen klinische Verschlechterungen frühzeitig erkennen helfen. Durch KI-gestützte Anwendungen für Routineuntersuchungen oder Schulungen könnten Belastungen zumindest teilweise verringert werden. Es sollte im Sinne der Partizipation herausgefunden werden, was die Bedürfnisse und Präferenzen der Patient:innen hinsichtlich einer Unterstützung durch KI sind.

Dieser Übersichtsartikel fasst den aktuellen Forschungsstand zum Einsatz von KI in verschiedenen Bereichen der Kinder- und Jugendmedizin und der Kinder- und Jugendpsychiatrie zusammen. Anhand häufiger Krankheitsbilder und Entwicklungsstörungen werden Anwendungsmöglichkeiten dargestellt, die Perspektiven verschiedener Stakeholder auf eine potenzielle Implementierung in die klinische Praxis beleuchtet und die Chancen und Risiken einer zukünftigen Umsetzung kritisch diskutiert. Die Grundlage für die Auswahl der Artikel bilden 2 systematische Übersichtsarbeiten unserer Arbeitsgruppe [7, 8]. Diese wurden durch eine erneute, gezielte Medline-Suche im Januar 2025 mit den Suchtermen ((artificial intelligence*) OR (machine learning*) OR (deep learning*) OR (neural networks*)) AND (((chronic disease*) OR ((development) OR (health))) AND ((child*) OR (adolescen*))) und um eine Einordnung der Akzeptanz der möglichen Anwendungen ergänzt. Die Auswahl der Beispiele für konkrete Störungen und Krankheitsbilder erfolgte anhand deren Häufigkeit sowie der Anzahl der Studien zum entsprechenden Thema. Bisher fehlen Studien, die die tatsächliche Anwendung im klinischen Alltag untersuchen. Tabelle S1 (im Onlinematerial) gibt eine detaillierte Übersicht über die im Artikel erwähnten Studien.

## KI in der Entwicklungsbeobachtung von Kindern und Jugendlichen

Sowohl die Anwendungsbereiche als auch die Datenquellen und die ausgegebenen Daten bei KI-Anwendungen zur Entwicklungsbeobachtung und -diagnostik sind äußerst heterogen. In verschiedenen Studien werden Daten aus elektronischen Patient:innenakten, Videos aus dem häuslichen Umfeld, Audioanalysen von Sprachaufnahmen, aus Computerspielen, aus Fragebögen oder Sensordaten von Wearables verwendet [7, 9–14]. Es gibt einerseits Ansätze, die anhand von *Big-Data*-Analysen beispielsweise von medizinischen Registerdaten Faktoren für ein gesundes Aufwachsen identifizieren [10], und andererseits solche, die individuelle Daten aus Eingaben bei einem Computerspiel mit individuellen Diagnosen oder Prognosen verknüpfen [13, 15].

Die Zielgruppe solcher KI-Anwendungen sind ebenfalls unterschiedlich, teils richten sie sich an medizinisches Fachpersonal, teils an Eltern. Hieraus ergeben sich unterschiedliche Anforderungen an die Anwendung und vor allem an die Empfehlungen, die diese ausspricht. Während medizinisches Fachpersonal die Informationen in den Kontext seines Wissens und Erfahrungsschatzes einordnet und die Familien entsprechend beraten kann, ist dies für medizinische Laien in der Regel deutlich schwieriger.

Bisher gibt es kaum Forschungsarbeiten, in denen die tatsächliche Anwendbarkeit im klinischen Alltag oder die langfristigen Outcomes untersucht wurden. Dennoch zeigen die bisherigen Ansätze, in welchen Bereichen künftig KI-gestützt eine Verbesserung der Versorgung von Kindern und Jugendlichen zu erwarten ist.

### Entwicklungsstörungen und -verzögerungen

Für die Beurteilung der allgemeinen Entwicklung von Kindern gibt es verschiedene Ansätze, die in der Regel spezifische Aspekte der Entwicklung fokussieren. Die feinmotorische Entwicklung wurde in einer Studie beispielsweise durch die Analyse gemalter Bilder beurteilt, wobei auch im weiteren Sinne die Schulfähigkeit überprüft wurde. Durch die Analyse wurden den Eltern spezifische Förderbereiche für ihr Kind vorgeschlagen [16]. Durch eine automatisierte Ganganalyse mittels plantarer Druckmessung können Kinder mit Erkrankungen des Bewegungsapparates früher eine adäquate Therapie erhalten [17]. Durch das Tragen von Bewegungssensoren oder die Auswertung einfacher Aktivitäten wie das Werfen eines Balles können Bewegungsauffälligkeiten bei Kindern mit Risiko für motorische Entwicklungsverzögerungen detektiert werden [18, 19].

Auch ein durch Lehrer:innen durchgeführtes KI-gestütztes Screening hinsichtlich sozialer oder Verhaltensauffälligkeiten war in einer Studie erfolgreich [20]. Eine in Indien durchgeführte Studie konnte KI-gestützt anhand verschiedener anthropometrischer Daten und solcher aus verschiedenen Entwicklungstests den kognitiven Entwicklungsstand im Alter von 3 Jahren bei 12 Monate alten Kindern voraussagen [21], eine andere Studie anhand klinischer Daten den Entwicklungsstand sehr kleiner Frühgeborener im Alter von 2 Jahren [22].

Mehrere Studien konnten anhand von Daten, die durch die Eltern eingegeben wurden, Kinder mit einer auffälligen Entwicklung in verschiedenen Bereichen erkennen [23, 24]. Für Ärzt:innen könnte die Diagnosestellung einer Sprachentwicklungsverzögerung durch ein KI-gestütztes *Decision Support Tool* einfacher werden [25].

### Autismus-Spektrum-Störung

Zahlreiche Studien beschäftigen sich mit der Diagnosestellung von Autismus-Spektrum-Störungen (ASS), deren Prävalenz insbesondere in den USA in den letzten Jahren deutlich zugenommen hat [26]. Dementsprechend vielfältig sind auch die methodischen Ansätze.

Die KI-gestützte Auswertung von im häuslichen Umfeld erhobenen Daten ist ein größeres Forschungsfeld, das in Studien erste Erfolge zeigte. Die Datenquellen sind dabei Videos oder Sprachaufnahmen, die entweder spezifisch für die Studie erhoben oder im Alltag aufgezeichnet und später genutzt wurden [12, 27, 28]. Eine KI, die zur Auswertung von Handgesten auf standardisiert aufgenommenen Fotos trainiert wurde, konnte in einer kleinen Kohorte typische Muster erkennen [29]. Weitere Arbeiten untersuchen das Spielverhalten von Kindern mit ASS und leiten daraus typische Reaktions- und Bewegungsmuster ab [30, 31]. Auch spezifischere Untersuchungen wie Augenbewegungsmuster oder Elektroenzephalogramme können KI-gestützt ausgewertet werden und Hinweise auf eine ASS geben [32, 33].

Da auch bei ASS die frühzeitige Diagnosestellung und Beginn einer psychotherapeutischen Behandlung sowie Fördermaßnahmen eine wesentliche Rolle für das Outcome spielen, beschäftigen sich einige Studien mit der Voraussage des Erkrankungsrisikos. Eine Arbeit untersuchte die elektronischen Patient:innenakten mehrerer Tausend Elternpaare von Kindern mit und ohne ASS und konnte Risikofaktoren wie Medikamenteneinnahme, hormonelle und ernährungsbedingte Einflüsse sowie den elterlichen Altersunterschied identifizieren [34]. In einer anderen Studie wurden die elektronischen Akten kleiner Kinder ausgewertet, um frühzeitig Patient:innen mit Risiko zu erkennen. Hierbei wurden das Vorliegen eines Neugeborenenikterus und das Vorhandensein von Familienangehörigen mit Entwicklungsauffälligkeiten als Risikofaktoren identifiziert [35].

Auch bei der Auswertung psychologischer Fragebögen, Tests und Skalen erwies sich KI in einigen Studien als hilfreich. Perspektivisch könnte sie durch automatisierte Auswertung und die Alarmierung bei Auffälligkeiten eine Unterstützung für testendes Fachpersonal bieten. Zudem könnte die Verkürzung der bestehenden Fragebögen mithilfe von KI-Methoden möglich sein, da einige Studien nur ausgewählte Items für die Diagnosestellung benötigten [36–40].

### Lese-Rechtschreib-Störungen

Verschiedene Studien untersuchen die niedrigschwellige Detektion einer umschriebenen Entwicklungsstörung schulischer Fertigkeiten, wie z. B. einer Lese-Rechtschreib-Störung (LRS) oder einer Rechenstörung. Diese Verdachtsdiagnose kann über ein Web-Spiel, das Kinder und Jugendliche spielen sollen, über Textnachrichten oder über die Tonaufnahme von Sprache gestellt werden [13, 15]. Auch standardisierte Tests zur sprachlichen und kommunikativen Entwicklung können durch KI-gestützte Auswertung schon im Kleinkindalter Kinder mit einem erhöhten Risiko für eine LRS erkennen [14]. Die Umsetzung derartiger niederschwelliger Testverfahren könnte ein wichtiger Baustein in der zukünftigen früheren Förderung von Kindern mit LRS sein, die am effektivsten wirkt, wenn die Störung bereits vor dem Eintritt in die Grundschule erkannt wird. Dies ist aktuell jedoch meist nicht der Fall. Durch die frühzeitige Diagnose könnten Folgeprobleme wie generelle Schulschwierigkeiten und psychiatrische Co-Morbiditäten verhindert oder besser behandelt werden [41].

### Mentale Gesundheit

Nach der Covid-19-Pandemie bleibt die Zahl der Kinder und vor allem Jugendlichen, die an einer psychischen Erkrankung leiden, hoch [42]. Suizid war 2023 die häufigste Todesursache bei Jugendlichen und jungen Erwachsenen [43]. Eine bessere Prävention in diesem Bereich ist also dringend notwendig. In 2 Studien zeigte die Analyse von Online-Posts erste Erfolg versprechende Ergebnisse. Durch die KI-gestützte Analyse von Beiträgen und Reaktionen in einer Diabetesgruppe auf Facebook konnte die psychische Belastung durch die Erkrankung bei einzelnen Individuen erkannt werden [44]. Ein Screening auf Suizidalität anhand von Beiträgen auf der Online-Diskussionsplattform Reddit, basierend auf zuvor definierten *At-Risk-*Wörtern und -Verhaltensweisen, war ebenfalls erfolgreich [45]. Diese Arbeiten zeigen einen Ansatz auf, wie durch die Nutzung neuer Datenquellen Kinder und Jugendliche mit einem Risiko für psychische Erkrankungen identifiziert werden können, die ansonsten möglicherweise keine ärztliche oder psychologische Hilfe in Anspruch nehmen würden. Dennoch muss hier das Recht auf Privatsphäre und Schutz der eigenen Daten kritisch diskutiert und mit dem potenziellen Nutzen ins Verhältnis gesetzt werden [46].

## KI in der Betreuung von Kindern und Jugendlichen mit chronischen Erkrankungen

Bei der Betreuung von Kindern und Jugendlichen, bei denen chronische Erkrankungen bereits diagnostiziert wurden und die daher eine regelmäßige medizinische Betreuung brauchen, gibt es vielfältige KI-gestützte Systeme, die die Kinder und Jugendlichen selbst, ihre Familien oder die betreuenden medizinischen Teams unterstützen können. Viele Arbeiten befassen sich mit der Prognose verschiedener Erkrankungen, wodurch die Familien besser beraten werden können, oder entwickeln und testen Entscheidungsunterstützungssysteme (*Decision Support Tools*), die medizinisches Personal bei der Entscheidungsfindung und der Behandlung der Patient:innen unterstützen. Darüber hinaus werden Anwendungen, die die Leitlinienadhärenz medizinischen Personals unterstützen, entwickelt [8].

### Diabetes mellitus Typ 1

*Hybrid-Closed-Loop*-Insulinpumpen sind das prominenteste Beispiel einer KI-gestützten Technologie, die bereits in der klinischen Anwendung ist [47]. Die automatisierte Abgabe von Insulin durch die mit einem Glukosesensor verbundene Pumpe ist heute eine leitliniengerechte Therapie (AWMF-LL 2024) und kann den Anteil der Zeit im Zielbereich (*Time in Range*) im Vergleich zu anderen Therapieformen erhöhen. Damit wird das Risiko für Diabetesfolgeerkrankungen bei betroffenen Kindern und Jugendlichen deutlich reduziert. Derzeit ist es bei diesen sogenannten Automatische-Insulinabgabe-Systemen (*Automated Insulin Delivery Systems*, AID) noch notwendig, dem System die Kohlenhydrataufnahme mitzuteilen. Hier gibt es jedoch auch schon erste Ansätze, die Kohlenhydratmenge einer Mahlzeit per Handyfoto automatisiert zu entwickeln [48]. In der Betreuung von Patient:innen mit Diabetes können zudem Roboter eingesetzt werden, die verschiedene Rollen einnehmen können: Sie können eine spielerische Beziehung zu den Patient:innen aufbauen und dadurch zum Beispiel zur Motivation oder zum emotionalen Wohlbefinden beitragen und eine Art freundschaftlicher Krankheitsbegleiter sein; sie können die Kinder und Jugendlichen in der Krankheitsbewältigung unterstützen, die Gesundheitskompetenz (*Health Literacy*) verbessern und Copingstrategien vermitteln; sie können die Ernährung und das Krankheitsmanagement unterstützen [49]. Solche Roboter wurden in ersten Studien gut von den Kindern akzeptiert und die Interaktion mit ihnen wurde positiv bewertet [50, 51]. Hier müssen sicherlich Langzeitdaten zur Wirkung abgewartet werden und die Übernahme einer Ratgeberfunktion für Kinder durch eine Maschine wird insbesondere vor dem Hintergrund der vielen Zeit, die Kinder mit digitalen Medien verbringen, auch kritisch gesehen [52].

## Akzeptanz von KI in der Kinder- und Jugendmedizin

### Akzeptanz durch medizinisches Personal.

**Abb. 1:**
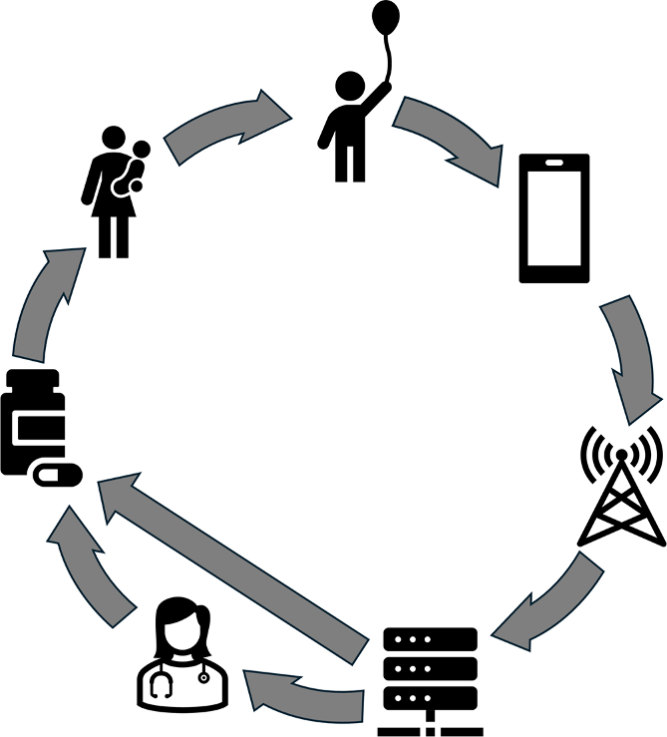
*KI-gestützte Prävention und Krankheitsbegleitung von Kindern und Jugendlichen. *In diesem Beispiel wird ein Kind mit einer chronischen Erkrankung regelmäßig in seinem Alltag mithilfe eines mobilen Endgerätes überwacht. Die Daten werden dann zur weiteren Analyse übertragen und ausgewertet. Eine Rückmeldung über eine möglicherweise notwendige Therapieänderung wird dann entweder zunächst von einer Ärztin bzw. einem Arzt überprüft (sog. Human-in-the-loop-Konzept) oder direkt an die Eltern übermittelt. Der Erfolg der Therapieumstellung wird dann sofort wieder durch die Überwachung und Analyse überprüft

Wie in allen befragten Gruppen ist die Akzeptanz von KI durch medizinisches Personal insgesamt hoch. Ärzt:innen erhoffen sich u. a. durch die KI-gestützte Entscheidungsfindung eine Verbesserung der Behandlungsqualität und Patient:innensicherheit [53], aber auch mehr Zeit für die Behandlung und Betreuung von Kindern und Jugendlichen und ihrer Familien durch die Übernahme administrativer Tätigkeiten durch KI [54, 55]. Für sie sind die Sicherstellung des Datenschutzes sowie die Nutzung hochwertiger und verlässlicher Daten beim Training von KI-Algorithmen sehr relevant [53]. Medizinisches Fachpersonal betonte die Wichtigkeit von Schulungen zum Umgang mit KI-Systemen sowie die Bedeutung eines *Human-in-the-Loop-Ansatzes*, also der menschlichen Kontrolle von KI-gestützten Entscheidungen ([53]; Abb. 1). Aus ethischer Sicht wird zudem regelmäßig auf Probleme wie Diskriminierung durch verzerrte Trainingsdaten und ein Schädigungspotenzial aufgrund – zumindest bislang – unzureichender klinischer Testung hingewiesen. Speziell bei Jugendlichen stellt sich außerdem die Frage, wie das Recht auf informationelle Selbstbestimmung angemessen respektiert werden kann [56, 57].

### Akzeptanz durch Eltern.

Eltern haben die Hoffnung, dass durch den Einsatz von KI Diagnosen schneller gestellt und die Früherkennung von Krankheiten verbessert werden kann. Sie wünschen sich zudem individualisierte Entscheidungen und Behandlungspläne, die KI-gestützt generiert werden könnten [58]. Sie hoffen, dass dem medizinischen Personal durch einen Einsatz von KI für administrative Aufgaben mehr Zeit für den persönlichen Kontakt zu Patient:innen und Familien bleibt. Wie auch medizinisches Personal und Kinder und Jugendliche selbst betonen sie, dass die persönliche Beziehung zum Behandlungsteam durch den Einsatz von KI keinesfalls in Mitleidenschaft gezogen werden dürfe [54, 55]. Sie fürchten Fehlentscheidungen von Algorithmen und die Verantwortungszuschreibung für KI-gestützte Entscheidungen, die sie selbst nicht vollständig nachvollziehen können [53]. Eltern möchten über einen möglichen Einsatz von KI in der Behandlung ihrer Kinder aufgeklärt und an der Entscheidungsfindung, ob KI genutzt wird, beteiligt werden und die Möglichkeit haben, den Einsatz abzulehnen. Dies könnte sich organisatorisch jedoch als schwierig oder gar nicht umsetzbar erweisen, wenn ein Krankenhaus oder eine Arztpraxis auf ein KI-System umgestellt hat. Die Eltern fordern, dass die Vorteile eines Einsatzes von KI allen Kindern und Jugendlichen unabhängig von ihrem sozioökonomischen und Versicherungsstatus zugutekommen sollten. Eine weitere Befürchtung ist ein zu großes Vertrauen von Ärzt:innen in KI-Systeme und dadurch zustande kommende Fehldiagnosen oder eine schlechtere Behandlung ihrer Kinder [55, 58, 59].

### Akzeptanz durch Kinder und Jugendliche.

Bisher gibt es wenige Studien, die die Akzeptanz von KI durch Kinder und Jugendliche untersuchen. Kinder und Jugendliche gaben in einer Studie zur KI-gestützten Auswertung von Röntgenbildern an, sich eine Verbesserung der Genauigkeit und Reduktion von Fehl- oder übersehenen Diagnosen zu erhoffen [60]. Wie auch Eltern und medizinisches Personal erwarten Kinder und Jugendliche, dass Ärzt:innen durch eine KI-Unterstützung bei administrativen Aufgaben und Dokumentation mehr Zeit für Patient:innen und deren Familien bleibt, befürchten aber andererseits, dass es durch den Einsatz von KI zu einem Verlust der persönlichen Interaktion und Beziehung zwischen Behandlungsteam und Familien kommen könnte [54, 55].

## Fazit

Der Einsatz von KI in der Kinder- und Jugendmedizin und -psychiatrie steht noch am Anfang und findet sich eher in wissenschaftlichen Projekten als im Praxisalltag. Er birgt jedoch ein enormes Potenzial, sowohl in der Prävention als auch in der Betreuung von Kindern und Jugendlichen mit chronischen Erkrankungen. Insbesondere bei der frühzeitigen Erkennung von Entwicklungsstörungen und Erkrankungen wie LRS oder Autismus-Spektrum-Störungen sowie bei der kontinuierlichen Überwachung und Unterstützung chronisch erkrankter Kinder können KI-gestützte Anwendungen wertvolle Beiträge leisten. Sie könnten in Zukunft präzisere Diagnosen, individualisierte Therapieansätze und eine Entlastung medizinischer Fachkräfte ermöglichen.

Jedoch stehen diesem Potenzial auch erhebliche Herausforderungen gegenüber. Die Sicherheit bei Entscheidungen und der Schutz sensibler Daten, die Transparenz der eingesetzten Algorithmen sowie die breite Akzeptanz der Technologie durch alle beteiligten Stakeholder – von den Kindern und Jugendlichen über ihre Eltern bis hin zu medizinischem Personal – sind entscheidende Faktoren für eine erfolgreiche Implementierung. Sorgen hinsichtlich eines Verlustes persönlicher Interaktionen, möglicher Fehlentscheidungen durch KI-Systeme sowie eines Missbrauchs der Daten müssen adressiert werden.

Zudem können KI-Systeme nur dann präzise Aussagen treffen, wenn die zugrunde liegenden Daten repräsentativ für die entsprechende Gruppe sind. Insbesondere in der Kinder- und Jugendmedizin sind umfangreiche altersdifferenzierte Datensätze von Kindern und Jugendlichen erforderlich, die frei von Verzerrungen (Bias) sind – etwa solche aus großen Universitätskliniken oder bestimmten geografischen Regionen. Systeme, die für Erwachsene mit Daten von erwachsenen Patient:innen entwickelt wurden, dürfen in der Kinder- und Jugendmedizin erst nach rigoroser Prüfung und Validierung für diese Patient:innengruppe eingesetzt werden.

Entwicklung und Nutzung von KI-Anwendungen in der Kinder- und Jugendmedizin sollten darauf abzielen, individuelle Vorteile und Verbesserungen in der Versorgung gleichermaßen zu fördern. Hierfür bedarf es weiterer Forschung zur klinischen Machbarkeit und zum tatsächlichen Nutzen in der Versorgung. Sie muss auch die ethischen, sozialen und rechtlichen Aspekte berücksichtigen. Durch einen verantwortungsvollen und partizipativen Ansatz bei der Entwicklung von Anwendungen kann KI dazu beitragen, die Versorgung von Kindern und Jugendlichen nachhaltig zu verbessern (s. auch Infobox 1).

### Infobox Das Projekt AI-PHCA

Das Projekt AI-PHCA (Artificial Intelligence in Preventive Health Care for Children and Adolescents) untersucht, wie künstliche Intelligenz (KI) genutzt werden kann, um die präventive Gesundheitsversorgung von Kindern und Jugendlichen zu verbessern. Dabei werden KI-basierte Ansätze wie Smartphone-Apps und webbasierte Anwendungen aus ethischer, rechtlicher und sozialer Perspektive analysiert. Ein zentrales Ziel ist es, die Einstellungen von Kindern, Jugendlichen, Eltern sowie Kinderärztinnen und -ärzten zum Einsatz solcher KI-Tools zu erfassen und die damit verbundenen ethischen und rechtlichen Herausforderungen zu identifizieren. Das interdisziplinäre Projekt zielt darauf ab, eine fundierte Datenbasis und umfassende Analysen bereitzustellen, um die verantwortungsvolle Integration von KI in die pädiatrische Präventivmedizin zu fördern.

Weitere Informationen: https://www.ai-phca.de/.

## Supplementary Information

Tabelle S1: Übersicht über die zitierten Studien
